# Low expression of long noncoding RNA PANDAR predicts a poor prognosis of non-small cell lung cancer and affects cell apoptosis by regulating Bcl-2

**DOI:** 10.1038/cddis.2015.30

**Published:** 2015-02-26

**Authors:** L Han, E-b Zhang, D-d Yin, R Kong, T-p Xu, W-m Chen, R Xia, Y-q Shu, W De

**Affiliations:** 1 Department of Oncology, First Affiliated Hospital of Nanjing Medical University, Nanjing, Jiangsu, PR China; 2 Department of Biochemistry and Molecular Biology, Nanjing Medical University, Nanjing, Jiangsu, PR China; 3 Cancer Research and Therapy Center, The Second Affiliated Hospital of Southeast University, Nanjing, Jiangsu, PR China; 4 Laboratory Center, Subei People's Hospital, Yangzhou, Jiangsu, PR China

## Abstract

Recently, a novel class of transcripts, long noncoding RNAs (lncRNAs), is involved in diseases including cancer. Here, we investigated the the role of lncRNA PANDAR in the progression of non-small cell lung carcinoma (NSCLC). PANDAR, interacting with NF-YA, was generally downregulated in NSCLC tissues. In a cohort of 140 NSCLC patients, decreased PANDAR expression was negatively correlated with greater tumor size (*P*<0.001) and advanced TNM stage (*P*=0.002). Moreover, PANDAR could serve as an independent predictor for overall survival in NSCLC (*P*=0.015). Further experiments demonstrated that PANDAR expression was induced by p53, and chromatin immunoprecipitation (ChIP) assays confirmed that PANDAR was a direct transcriptional target of p53 in NSCLC cells. PANDAR overexpression significantly repressed the proliferation *in vitro* and *in vivo*. We also showed that PANDAR-mediated growth regulation is in part due to the transcriptional modulation of Bcl-2 by interacting with NF-YA, thus affecting NSCLC cell apoptosis. To our knowledge, this is the first report which showed the role of PANDAR in the progression of NSCLC. The p53/PANDAR/NF-YA/Bcl-2 interaction might serve as targets for NSCLC diagnosis and therapy.

Lung cancer is the most frequent cause of cancer-related death all over the world, in which non-small cell lung cancer (NSCLC) accounts for about 80–85% of all lung cancer cases. Although in recent years there are mounting progresses in clinical treatment for NSCLC, the overall survival (OS) time of NSCLC patients has not improved dramatically. An important reason for that is the lack of molecular biomarkers, and therefore most of NSCLC are diagnosed at an advanced stage.^1^ Thus, disclosing the molecular mechanism for NSCLC is necessary to diagnose and develop effective therapy at the earliest.

Formerly, the investigation of mechanisms of tumorigenesis mainly focused on protein-coding genes. Recently, long noncoding RNAs (lncRNAs) have attracted our major attention. The lncRNAs are important new members of the ncRNA family, which are greater than 200 nt, and are unable to be translated into proteins. So far, many lncRNAs are known to play important roles in cellular development, differentiation, immune response and many other biological processes.^2, 3, 4, 5, 6, 7, 8, 9^ These findings demonstrate that lncRNAs display a major role in the regulation of the eukaryotic genome. In addition, the disregulation of lncRNAs has been shown in various types of cancers, including NSCLC.^10, 11, 12, 13^

The molecular mechanisms of lncRNAs are diverse. Mounting evidences revealed that lncRNAs could play an important role in regulating gene expression at different levels, including chromatin modification, transcriptional and posttranscriptional processing.^14, 15^ For instance, overexpression of HOTAIR could promote breast cancer metastasis by reprograming the chromatin state.^10^ Linc-MD1 can control muscle differentiation by serving as a ‘sponge' to titrate microRNAs.^16^

Recently, numerous lncRNAs have been identified to function by interacting with RNA-binding proteins, such as hnRNP, SUZ12 and NF-YA, thus regulating gene expression.^10, 17, 18, 19^ LncRNA PANDAR (promoter of CDKN1A antisense DNA damage-activated RNA) was firstly reported by Hung *et al.*^20^ They found that DNA damage could induce five lncRNAs from the CDKN1A promoter, and one such lncRNA, named PANDAR, is induced in a p53-dependent manner. Moreover, PANDAR interacts with the transcription factor NF-YA to limit the expression of pro-apoptotic genes. The above findings were based on normal human fetal lung fibroblasts. As we all know, DNA damage is closely associated with tumorigenesis. In addition, NF-YA is closely related to tumorigenesis;^21, 22^ and the role of PANDAR in lung cancer has not been investigated. These prompted us to explore the role of PANDAR in human NSCLC.

In the present study, we found that lncRNA PANDAR was significantly downregulated in NSCLC tissues than that in corresponding non-tumor lung tissues. Decreased PANDAR expression was negatively correlated with greater tumor size (*P*<0.001) and advanced TNM stage (*P*=0.002). Moreover, PANDAR could be served as an independent predictor for OS in NSCLC (*P*=0.015). Furthermore, PANDAR was a direct transcriptional target of p53 and could regulate cell growth both *in vitro* and *in vivo*. Furthermore, we demonstrated that PANDAR could modulate the BCL2 by binding to NF-YA, which may partly account for PANDAR-mediated apoptosis regulation, thus affecting the proliferation of NSCLC both *in vitro* and *in vivo*.

## Results

### PANDAR is downregulated in human NSCLC tissues and negatively correlated with tumor size and advanced TNM stage

**Figure 1:**
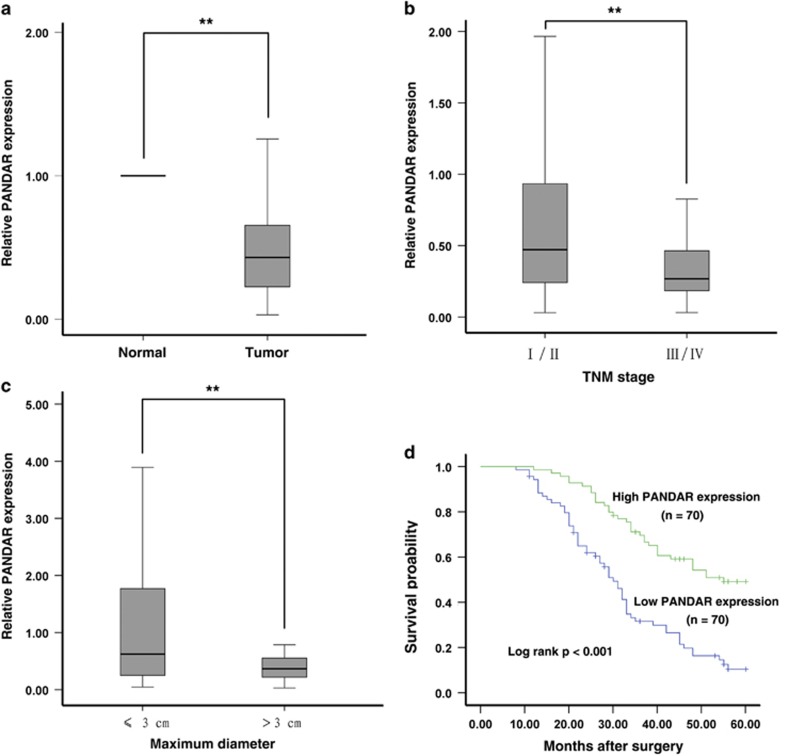
Analysis of PANDAR expression in NSCLC tissues and clinical parameters. (**a**) PANDAR was detected in 140 pairs of NSCLC tissues by qRT-PCR and normalized to GAPDH expression. The levels of PANDAR in NSCLC tissues are significantly lower than those in non-tumorous tissues. (**b**) and (**c**) PANDAR expression was significantly lower in patients with a higher pathological stage and big tumor size. (**d**) Patients with low levels of PANDAR expression showed reduced survival times compared with patients with high expression (*P*<0.001, log-rank test). **P<0.01

PANDAR expression levels were investigated by qRT-PCR. Among all 140 pairs of NSCLC patients, PANDAR expression level was significantly downregulated in 82.9% (116 in 140) of tumors than those in the corresponding normal tissue (Figure 1a). Next, we examined the correlation of PANDAR expression level with the clinico-pathological factors in NSCLC. As shown in Figures 1b and c, there was an obvious inverse correlation between decreased PANDAR levels and advanced TNM stage (0.9087±1.07593 *versus* 0.3813±0.34864, *P*=0.002) and greater tumor size (1.2433±1.32007 *versus* 0.5136±0.58505, *P*<0.001).

### Decreased expression of PANDAR is associated poor prognosis of NSCLC

To determine the relationship between PANDAR expression and NSCLC patients' prognosis, we attempted to evaluate the correlation between PANDAR expression and clinical outcomes. Kaplan–Meier survival analysis and log-rank tests were performed to evaluate the correlation between PANDAR expression and the prognosis of patients with NSCLC. We firstly divided the samples into high (above the mean, *n*=70) and low (below the mean, *n*=70) PANDAR expression groups according to the mean expression level of PANDAR. The results showed that 5 years of OS for high PANDAR expression groups was 32.8%, whereas that for low PANDAR expression groups was only 5.7%. The median survival time for high PANDAR expression groups was 46.749±1.831 months, whereas that for low PANDAR expression groups was only 32.437±1.871 months. As shown in Figure 1d, decreased PANDAR level was associated with shorter OS (*P*<0.001).

To further confirm the prognostic role of PANDAR in NSCLC patients, the univariate and multivariate survival analysis (Cox proportional hazards regression model) were performed. Univariate analysis identified four prognostic factors: histological grade (low, middle or high), TNM stage (I/II, III/IV), lymph node metastasis (N0, N1 or above) and PANDAR expression. The other clinico-pathological characteristics, such as age (≤60, >60), sex (male, female), histological classification (SCC, AD or others), tumor size (≤3 cm, >3 cm) and history of smoking (ever, never) were found not to be statistically significant prognosis factors. Multivariate analysis further revealed that PANDAR expression was a significant independent predictor of poor survival in NSCLC patients (*P*=0.015), as well as histological classification (*P*=0.004) and TNM stage (*P* <0.001) (Table 1). From what has been discussed above, we concluded that downregulated PANDAR might play an important role in the development of NSCLC.

### PANDAR is induced by p53, and p53 directly interacts with the p53 response element in the upstream region of PANDAR

**Figure 2:**
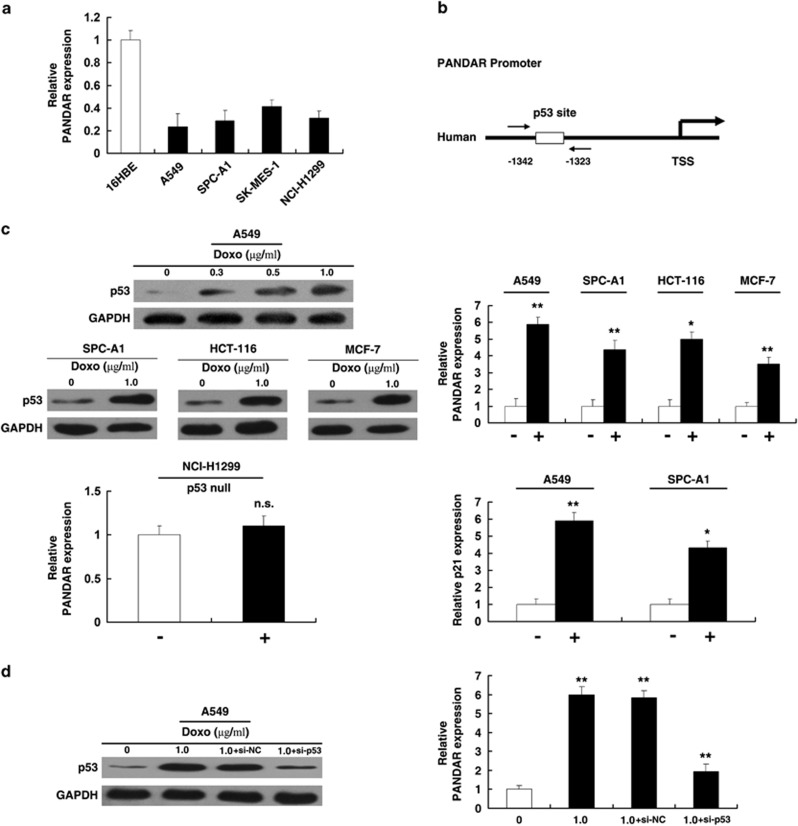
p53 induces the expression of PANDAR. (**a**) Analysis of PANDAR expression levels in NSCLC cell lines (A549, SPC-A1, SK-MES-1 and NCI-H1299) compared with the normal bronchial epithelial cell line (16HBE) by qRT-PCR. (**b**) Description of p53RE in promoter region of PANDAR. The position of ChIP primers was indicated by arrows. (**c**) Western blotting was used to detect the p53 induction by doxo in different cell lines. qRT-PCR was used to detect the effect of doxo on PANDAR expression in p53-WT and p53-null cells. (**d**) Western blotting and qRT-PCR were used to detect expression after treatments of doxorubicin-treated A549 cells following p53 siRNA. *P<0.05, **P<0.01

To probe into the mechanism of low expression of PANDAR in NSCLC, firstly, qRT-PCR was performed to detect the expression of PANDAR in NSCLC cell lines. As shown in Figure 2a, four cancer cell lines (A549, SPC-A1, SK-MES-1 and NCI-H1299) expressed lower levels of PANDAR compared with the normal bronchial epithelial cell line (16HBE). Then, we analyzed the promoter region of PANDAR and detected the presence of the p53-binding sites (wild type (WT)), as shown in Figure 2b. We assumed that p53 could regulate PANDAR expression at the transcriptional level. Next, we treated A549 cells expressing WT p53 (A549 WT) with different concentrations of doxorubicin (doxo), a known DNA-damaging agent. After 24 h, western blot analysis was used to detect the expression level of p53 and the results showed that doxo could induce p53 in a dose-dependent manner (Figure 2c). Next, we treated A549 cells at 1.0 *μ*g/ml for 24 h and found that doxo could induce PANDAR expression. Such induction was also detected in other cell lines expressing wild-type p53, which are, SPC-A1, HCT-116 and MCF-7 (Figure 2c). To further verify the specific influence of p53 on PANDAR, we treated NCI-H1299 cells (a p53-null cell line) with doxo (1.0 *μ*g/ml). The result showed that almost no induction was detected in p53-null cells (NCI-H1299) (Figure 2c). P21, a noted p53-regulated gene, as a positive control, was induced by doxo. To further confirm the effect of p53 on PANDAR expression, as shown in Figure 2d, in doxo-treated A549 cells following p53 siRNA, this co-transfection could obviously reverse doxo-induced p53 expression. In addition, this treatment could also significantly reverse doxo-induced PANDAR expression.

**Figure 3:**
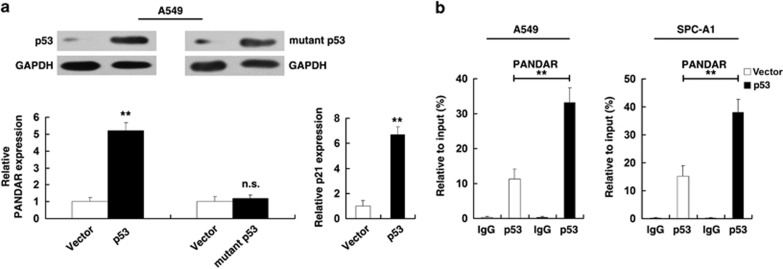
p53 could directly interact with the promoter region of PANDAR. (**a**) Induction of PANDAR by ectopically expressed p53 (wild-type p53 or mutant p53). Overexpression was confirmed by western blotting. (**b**) The p53 binding at the promoter regions of PANDAR was assessed by ChIP analysis. ChIP primers were detailed in Materials and Methods section. n.s., not significant

As doxo may induce cell response independent of p53, we enhanced p53 expression by transfecting a p53 expression vector (WT). The result also showed that enforced p53 expression could increase the expression of PANDAR, similar to the induction of a known p53-regulated gene, p21 (Figure 3a). Then, we sought to determine whether p53 mutation could regulate PANDAR expression. Toward this end, p53 with a point mutation (R175H) at the DNA-binding domain, a frequent mutant in diverse cancer,^23^ had no impact on PANDAR expression, indicating that PANDAR is specifically induced by WT p53. To determine whether p53 can directly bind to the sites of PANDAR promoter, ChIP-qPCR experiments were performed. As shown in Figure 3b, p53 immunoprecipitation was observed at the promoter of PANDAR in A549 and SPC-A1 cell lines. Overexpression of p53 could strengthen the p53 immunoprecipitation at the promoter of PANDAR. The position of ChIP primers was indicated by arrows (Figure 2b). An isotype-matched IgG was used as a negative control, p21 served as a positive control for ChIP assay (Supplementary Figure S1A).

Together, these results demonstrate that p53 interacts with the p53 response element in the PANDAR promoter, thus inducing the transcription of PANDAR.

### Effect of PANDAR on NSCLC cell growth *in vitro*

**Figure 4:**
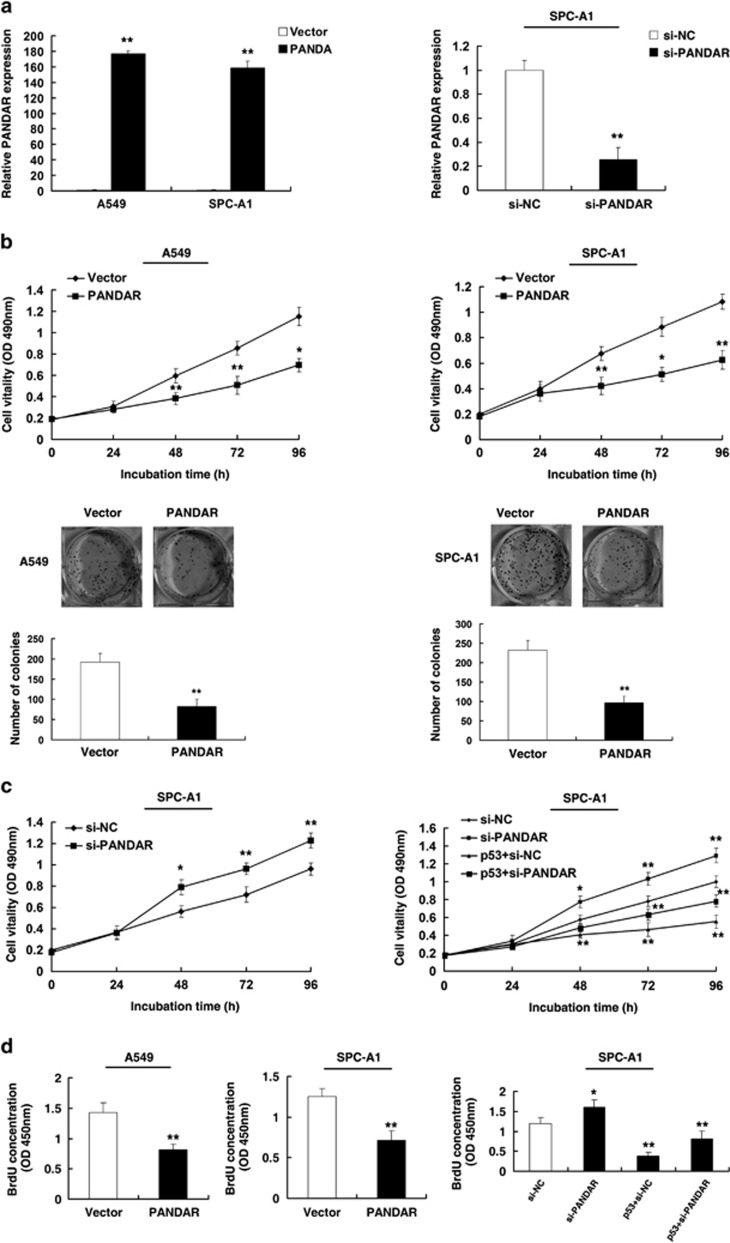
Effect of PANDAR on gastric cell growth *in vitro*. (**a**) The relative expression level of PANDAR in NSCLC cell lines, transfected with pCDNA-PANDAR and si-PANDAR, was tested by qPCR. (**b**) After transfection, MTT assay was performed to determine the proliferation of A549 and SPC-A1 cells. Representative results of colony formation of A549 and SPC-A1 cells transfected with empty vector or pCDNA-PANDAR. (**c**) After transfection with si-PANDAR, MTT assay was performed in SPC-A1 cell line. SPC-A1 transfected with si-NC/si-PANDAR/p53+si-NC and transfected with p53 followed by transfection with si-PANDAR. (**d**) BrdU assays were used to detect the cell proliferation after transfection, respectively. Error bars indicate means±S.E.M. *P<0.05, **P<0.01

To investigate the functional role of PANDAR in NSCLC cells, firstly, we examined the impact of PANDAR overexpression and knockdown in NSCLC cell lines. As shown in Figure 4a, qPCR assays showed that PANDAR expression was significantly induced both in A549 and SPC-A1 cell lines. And PANDAR expression was significantly reduced both in SPC-A1 cell line. After transfection, MTT assay showed that overexpression of PANDAR could significantly inhibit cell proliferation both in A549 and SPC-A1 cell lines compared with the control cells (Figure 4b). Similarly, the result of colony-formation assay revealed that clonogenic survival was significantly decreased following overexpression of PANDAR both in A549 and SPC-A1 cell lines (Figure 4b).

To further determine the physiological role of PANDAR in cells growth, SPC-A1 cells were transfected with p53 expression vector and followed by si-PANDAR. Then, MTT assays were performed. Our experiments showed that co-transfection of p53 and si-PANDAR could partly reverse p53-promoted growth arrest (Figure 4c). Moreover, BrdU assays also showed that overexpression of PANDAR could obviously repress cell proliferation both in A549 and SPC-A1 cell lines. And p53-mediated growth arrest was reversed when PANDAR expression was simultaneously downregulated (Figure 4d).

### PANDAR overexpression inhibits tumor growth of NSCLC cell *in vivo*

**Figure 5:**
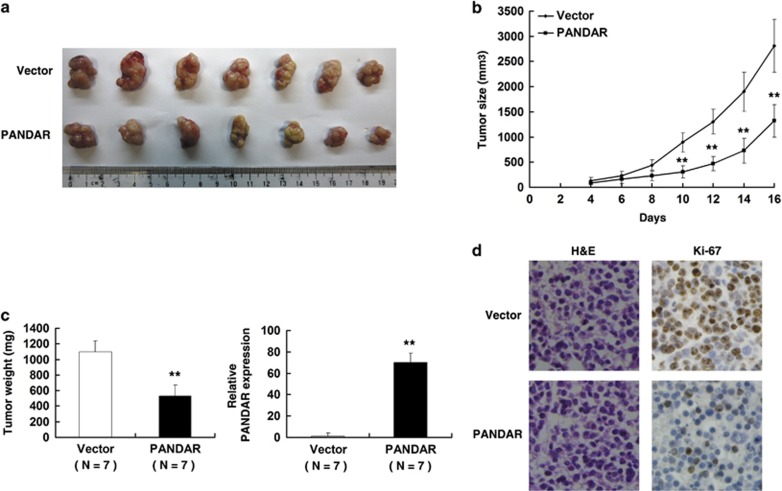
The impact of PANDAR on tumorigenesis *in vivo*. (**a**) and (**b**) Empty vector or pCDNA-PANDAR was transfected into A549 cells, which were injected in the nude mice (*n*=7), respectively. Tumor volumes were calculated after injection every 2 days. Bars indicate S.D. (**c**) Tumor weights are represented as means of tumor weights±S.D. qRT-PCR was performed to detect the average expression of PANDAR. (**d**) The tumor sections were under H&E staining and IHC staining using antibodies against Ki-67. Error bars indicate means±S.E.M. *P<0.05, **P<0.01

To confirm whether the PANDAR affects tumorigenesis, pCDNA-PANDAR and empty vector transfected A549 cells were inoculated into male nude mice. All mice developed xenograft tumors at the injection site. As shown in Figures 5a and b, tumor growth in pCDNA-PANDAR group was significantly slower than that in the empty vector group. Up to 16 days after injection, the average tumor weight in the pCDNA-PANDAR group was obviously lower than in the empty vector group (Figure 5c). QRT-PCR analysis was used to detect the average expression of PANDAR in tumor tissues (Figure 5c). Results suggested that the average level of PANDAR in the pCDNA-PANDAR group was higher than in the control group. We also found that the tumors developed from pCDNA-PANDAR cells displayed lower Ki-67 staining than that in tumors formed by empty vector cells, as detected by IHC analysis (Figure 5d).

### PANDAR regulates NSCLC cell apoptosis

**Figure 6:**
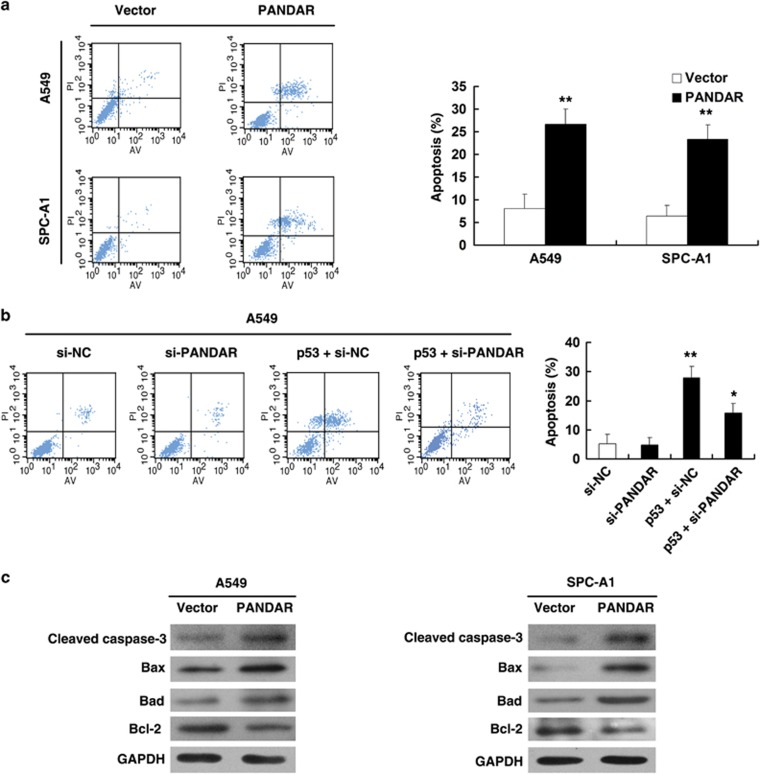
PANDAR regulates NSCLC cell apoptosis. (**a**) Forty-eight hours after transfection, A549 and SPC-A1 cells were stained and analyzed by flow cytometry. LR, early apoptotic cells. UR, terminal apoptotic cells. (**b**) A549 cells transfected with si-NC/si- PANDAR/p53+si-NC and transfected with p53 followed by transfection with si-PANDAR, Forty-eight hours after transfection, cells were stained and analyzed by flow cytometry. (**c**) Western blots analysis of activated caspase-3, Bcl-2 family proteins (Bax, Bad and Bcl-2) after empty vector or pCDNA-PANDAR transfection. GAPDH protein was used as an internal control

The above study shows that PANDAR plays a physiological role in regulating cell viability. To find out whether the effect of PANDAR on proliferation of NSCLC cells was by altering cell-cycle progression or apoptosis, flow cytometric analysis was performed. The result showed that the overexpression of PANDAR caused no significant changes in the percentages of cells in any of the cell-cycle phases (data not shown). Then, we examined the impact of PANDAR on apoptosis. As shown in Figure 6a, overexpression of PANDAR could greatly induce cell apoptosis both in A549 and SPC-A1 cell lines compared with the control cells. To further confirm the role of PANDAR in cells apoptosis, A549 cells were transfected with p53 expression vector and followed by si-PANDAR. After 48 h, cell apoptosis was analyzed by flow cytometric analysis. Our experiments showed that co-transfection of p53 and si-PANDAR could partly reverse p53-promoted apoptosis enhancement (Figure 6b).

To further explore the PANDAR-mediated apoptosis, we analyzed the expression of apoptosis-related proteins after transfection with pCDNA-PANDAR. The activation of caspase-3 could be observed after overexpression of PANDAR, both in A549 and SPC-A1 cell lines (Figure 6c). To elucidate the upstream pathway leading to caspase-3 activation, western blot analysis was used to detect the expression of bcl-2 protein family (Bax, Bad and Bcl-2). The results showed that PANDAR-overexpression could lead to increased levels of pro-apoptotic proteins (Bax and Bad) and decreased levels of anti-apoptotic protein (Bcl-2) (Figure 6c). Hence, it was concluded that ectopic PANDAR expression could induce caspase-3-dependent apoptosis in NSCLC cells.

### PANDAR could regulate Bcl-2 expression by interacting with NY-YA at the transcriptional level

**Figure 7:**
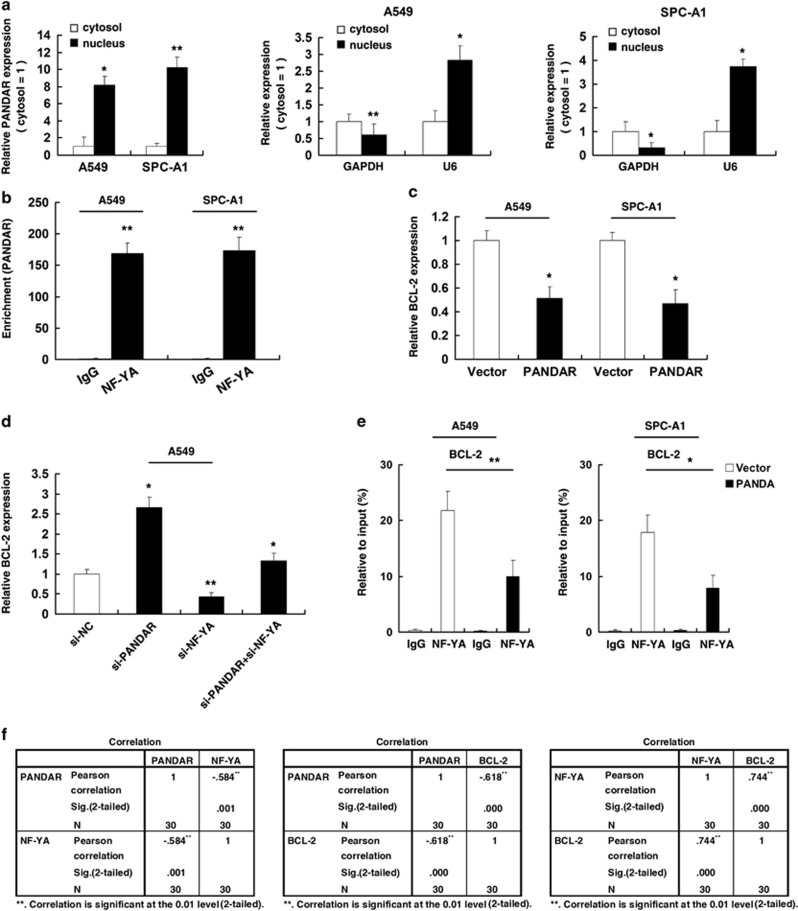
PANDAR could regulate Bcl-2 expression by interacting with NY-YA. (**a**) PANDAR nuclear localization, as identified using qRT-PCR in fractionated A549 and SPC-A1 cells. After nuclear and cytosolic separation, RNA expression levels in A549 and SPC-A1 cells were measured by qRT-PCR. GAPDH was used as a cytosol marker and U6 was used as a nucleus marker. (**b**) RIP experiments were performed in A549 and SPC-A1 cells and the coprecipitated RNA were subjected to qRT-PCR for PANDAR. The fold enrichment of PANDAR in NF-YA RIP is relative to its matching IgG control RIP. (**c**) and (**d**) qRT-PCR was performed to detect the expression of Bcl-2 genes in transfected cells. (**e**) After transfection with pcDNA-PANDAR, ChIP-qPCR was performed to detect the NF-YA binding at the promoter region of Bcl-2 locus. (**f**) The relationship between PANDAR-NF-YA-Bcl2 in NSCLC tissues

To further study the mechanism of its regulation of NSCLC cell apoptosis, firstly, we measured PANDAR expression in nuclear and cytosolic fractions from A549 and SPC-A1 cells by qRT-PCR. The differential enrichments of GAPDH and U6 RNA were used as fractionation indicators. We found a considerable increase in PANDAR expression in the nucleus *versus* the cytosol (Figure 7a), which suggests that PANDAR is mainly localized in the nucleus and may play a major regulatory function at the transcriptional level. In addition, Hung *et al.*^20^ found that PANDAR could interact with the transcription factor NF-YA in human fibroblasts. To examine whether PANDAR can interact with NF-YA in NSCLC cells, RNA immunoprecipitation (RIP) assays were performed. As shown in Figure 7b, the endogenous PANDAR was obviously enriched in the anti-NF-YA RIP fraction relative to the input compared with the IgG fraction both in A549 and SPC-A1 cell lines. The BCL-2 protein family determines the commitment of cells to apoptosis, especially Bcl-2, is upregulated in many types of tumors.^24^ In addition, Core promoters of cell apoptosis genes downstream of p53 are distinguished from other p53 target genes by the binding site for the transcription factor NF-YA.^25^ Moreover, NF-YA is closely related to tumorigenesis. Thus, we reasoned that PANDAR may affect NSCLC cell apoptosis through interacting with NY-YA, especially oncogene Bcl-2. Firstly, after overexpression of PANDAR, qRT-PCR was performed to detect the expression of Bcl-2. As shown in Figure 7c, overexpression of PANDAR could inhibit the expression of Bcl-2 both in A549 and SPC-A1 cell lines. And knockdown of PANDAR could induce the expression of Bcl-2 (Figure 7c). In addition, the knockdown of NF-YA could inhibit the expression of Bcl-2 (Figure 7c). To further determine the relationship between PANDAR-NF-YA-Bcl2, rescue experiments were performed. Firstly, A549 was transfected with siRNA targeting NF-YA, which efficiently reduced the NF-YA levels (Supplementary Figure S1B). Our experiments showed that co-transfection of si-PANDAR and si-NF-YA could partially abrogate si-PANDAR-induced Bcl-2 expression (Figure 7d). Moreover, chromatin immunoprecipitation (ChIP) results showed that overexpression of PANDAR could detect a loss of NF-YA binding at the promoter of Bcl-2 (Figure 7e). In an attempt to understand the relationship between PANDAR-NF-YA-Bcl2 in NSCLC tissues, we performed qRT-PCR analysis to detect the expression of PANDAR/NF-YA/Bcl2 in 30 pairs of NSCLC tissues. As shown in Figure 7f, PANDAR expression was significantly decreased in 30 pairs of NSCLC tissues, and NF-YA/Bcl2 was increased. Further analysis revealed that PANDAR expression was inversely correlated with NF-YA expression, PANDAR expression was inversely correlated with Bcl-2 expression, and NF-YA expression was positively correlated with Bcl-2 expression.

## Discussion

It has been widely accepted that mammalian genomes encode thousands of lncRNAs in addition to protein-coding RNAs.^26^ Although for each individual molecule, it needs to be further confirmed whether lncRNAs could execute important functions or just represent transcriptional noise or background transcription; for the majority of lncRNAs, their conservative evolution reveals that they might be functional.^26^ In addition to microRNAs, lncRNAs are emerging as important factors in cell biology. Thus far, increasing evidence links dysregulation of lncRNAs to diverse human diseases, including tumors.^27^

In our present study, we explored the correlation between PANDAR levels and clinico-pathological characteristics as well as the prognosis in patients with NSCLC. We found that the average level of PANDAR in NSCLC tissues was significantly lower than those in corresponding non-tumor tissues. Besides, we showed that decreased PANDAR expression was negatively correlated with greater tumor size and advanced TNM stage. It is more important that we also confirmed that PANDAR expression was an independent predictor for OS, as well as histological grade and TNM stage, which enhanced the clinical value of PANDAR. These results indicate that PANDAR may exhibit an important role in NSCLC development and progression.

Hung *et al.*^20^ found that DNA damage could induce PANDAR and p53 could also induce PANDAR in human fibroblasts. As we know, DNA damage is intimately associated with tumorigenesis. In addition, through bioinformation analysis, we found that the PANDAR promoter contained a conserved p53-binding site. And our results showed that wild-type p53 could induce PANDAR and indeed confirmed that PANDAR was a direct transcriptional target of p53 (WT). Owing to p53 mutations or absence accounting for up to 50% cancer cases, our study indicated that p53 may partly contribute to the downregulation of PANDAR in NSCLC.

Although PANDAR has been studied during DNA damage in human fibroblasts, a variety of physiological and pathological processes and the possible role of PANDAR in cancer including NSCLC remains to be clarified. In our study, the function of PANDAR was investigated by RNA interference (RNAi)-mediated knockdown and plasmid-induced overexpression. As a result, overexpression of PANDAR could obviously suppress NSCLC cell proliferation both *in vitro* and *in vivo*; and knockdown of PANDAR could promote NSCLC cell proliferation. In addition, p53-mediated growth arrest was found to be partly reversed by RNAi PANDAR. The growth inhibition induced by ectopic PANDAR overexpression might be associated with caspase-3-dependent apoptosis enhancement. Meanwhile, p53-mediated apoptosis induction was also found to be partly reversed by RNAi PANDAR. These results revealed that PANDAR may be an apoptosis-executor of p53 downstream.

The molecular mechanisms of lncRNAs are diverse. They could function (i) as decoys to locate transcription factors; (ii) as regulatory signals for transcription; (iii) as scaffolds to aggregate different proteins; (iv) as a ‘sponge' to interact with microRNAs and (v) as guides to binding to specific protein to target genes.^28^

We found a considerable increase in PANDAR expression in the nucleus, thus suggesting that PANDAR play a major regulatory function at the transcriptional level. In addition, Hung *et al.*^20^ found that PANDAR could interact with the transcription factor NF-YA in human fibroblasts. Our results showed that PANDAR could interact with NF-YA in NSCLC cell lines.

Bcl-2 protein family (Bcl-2, Bax and Bad) is the upstream pathway, which leads to caspase-3 activation. The Bcl-2 protein family determines the commitment of cells to apoptosis, especially Bcl-2, which is upregulated in many types of tumors.^24^ In addition, core promoters of cell apoptosis genes downstream of p53 are distinguished from other p53 target genes by the binding site for the transcription factor NF-YA.^25^ Moreover, NF-YA is closely related to tumorigenesis and can promote tumorigenesis.^22, 29^ We found that the overexpression of PANDAR could inhibit Bcl-2 at the transcriptional level. Moreover, co-transfection of si-PANDAR and si-NF-YA could partially abrogate si-PANDAR-induced Bcl-2 expression. ChIP assays confirmed that overexpression of PANDAR could cause a loss of NF-YA binding at the promoter of Bcl-2. Further analysis in NSCLC tissues revealed that PANDAR expression was inversely correlated with NF-YA expression, PANDAR expression was inversely correlated with Bcl-2 expression, and NF-YA expression was positively correlated with Bcl-2 expression. These results demonstrated that Bcl-2 was a *bona fide* target of PANDAR/NF-YA-regulated genes. Our findings provide a novel potential mechanism through which Bcl-2 boosts tumor cell proliferation in part due to the downregulation of lncRNA PANDAR, which releases the NF-YA.

Our study suggests that lncRNAs may also be a component of the p53-regulatory network, similar to protein-coding genes. For instance, lincRNA-p21, has been confirmed to be a p53 transcription target.^17^ Furthermore, we demonstrated that PANDAR-mediated promotion of NSCLC cell growth is at least in part through regulation of Bcl-2. Collectively, we showed that PANDAR is an important prognostic factor for NSCLC patients and regulates NSCLC cell proliferation both *in vitro* and *in vivo* bioassays. PANDAR/NF-YA-mediated regulation participates in the occurrence and development of NSCLC. Our study may supply a strategy for targeting the PANDAR/NF-YA/Bcl-2 interaction as a novel therapeutic application for NSCLC patients.

## Materials and Methods

### Tissue collection and Ethics statement

This study included 140 primary NSCLC patients who had undergone surgeries at First Affiliated Hospital of Nanjing Medical University between 2006 and 2007 (China). The clinico-pathological factors of patients are shown in Table 2. All patients did not receive chemotherapy or radiotherapy before surgery. All collected tissue samples were immediately snap-frozen in liquid nitrogen and stored until required. The study was approved by the Ethics Committee of Nanjing Medical University, and it was performed in compliance with the Declaration of Helsinki Principles; and each patient participated after providing informed consent. Patients discharged from hospital were followed up routinely according to a scheduled program, at least once a year.

### Cell culture

Human NSCLC adenocarcinomas cell lines (A549, SPC-A1 and NCI-H1299), a NSCLC squamous carcinomas cell line (SK-MES-1), a normal human bronchial epithelial cell line (16HBE), a colon cancer cell line (HCT-116) and a breast cancer cell line (MCF-7) were obtained from the Institute of Biochemistry and Cell Biology of the Chinese Academy of Sciences (Shanghai, China). Cells were cultured in RPMI 1640 or DMEM (GIBCO-BRL, Grand Island, NY, USA) medium supplemented with 10% FBS (Invitrogen, Grand Island, NY, USA), 100 U/ml penicillin, and 100 mg/ml streptomycin (Invitrogen) in incubator at 37 °C with 5% CO_2_.

### Reagents

Doxorubicin hydrochloride (Doxo) was purchased from Sigma (St. Louis, MO, USA).

### Transfection of cell lines

The PANDAR sequence was synthesized according to the full-length PANDAR sequence (based on the PANDAR sequence in NCBI) and then subcloned into a pCDNA3.1 vector (Invitrogen, Shanghai, China). The empty pcDNA3.1 vector was used as the control. p53 (WT) and mutant p53 (R175H) clone were purchased from Addgene (Cambridge, MA, USA). The plasmid was transfected by X-tremeGENE HP DNA transfection reagent (Roche, Basel, Switzerland), according to the manufacturer's instructions. siRNA for PANDAR: siRNA1# (5′-AAUGUGUGCACGUAACAGAUU-3′), siRNA2# (5′-GGGCAUGUUUUCACAGAGGUU-3′) and siRNA3# (5′-AAUGUGUGCACGUAACAGAUU-3′). The p53 siRNA was from Santa Cruz (Dallas, TX, USA; sc-29435). Non-specific siRNA (si-NC) was purchased from Invitrogen. Typically, cells were seeded at six-well plates and then transfected the next day with specific siRNA (100 nM) and control siRNA (100 nM) by using Lipofectamine RNAi MAX, according to the manufacturer's protocol (Invitrogen).

### RNA extraction and qRT-PCR analyses

Total RNA was extracted from tissues or cultured cells using TRIzol reagent (Invitrogen, Carlsbad, CA, USA). For qRT-PCR, RNA was reverse-transcribed to cDNA by using a Reverse Transcription Kit (Takara, Dalian, China). Real-time PCR analyses were performed with SYBR Green (Takara). Results were normalized to the expression of GAPDH. The qRT-PCR and data collection were carried out on ABI 7500 real-time PCR system (Applied Biosystems, Foster City, CA, USA). The primer sequences are summarized in Supplementary Table S1.

### Western blot assay

The cells were lysed using mammalian protein extraction reagent RIPA (Beyotime, Haimen, China) supplemented with protease inhibitors cocktail (Roche) and PMSF (Roche). Fifty micrograms of the protein extractions were separated by 10% SDS-PAGE transferred to 0.22 mm nitrocellulose membranes (Sigma-Aldrich, St. Louis, MO, USA) and incubated with specific antibodies.The autoradiograms were quantified by densitometry (Quantity One software; Bio-Rad, Hercules, CA, USA). Anti-p53 was from Santa Cruz Biotechnology (Dallas, TX, USA). Anti-cleaved caspase3, anti-Bax, anti-Bad and anti-Bcl-2 were from Cell Signaling Technology (Boston, MA, USA). Anti-NF-YA was from Abcam (Hong Kong, China). Results were normalized to the expression of GAPDH (Rabbit anti-GAPDH).

### Cell proliferation analysis

Cell viability was tested with MTT kit (Sigma) according to the manufacturer's instruction. For colony formation assay, a certain number of transfected cells were placed in each well of 6-well plates and maintained in proper media containing 10% FBS for 2 weeks, during which the medium was replaced every 4 days. Colonies were then fixed with methanol and stained with 0.1% crystal violet (Sigma) in PBS for 15 min. Colony formation was determined by counting the number of stained colonies. BrdU experiments were performed using a BrdU Cell Proliferation Assay Kit (Millipore, Billerica, MA, USA; Cat.No.2750) according to the manufacturer's instructions. The higher OD reading represents the higher BrdU concentration in the sample.

### Flow-cytometric analysis

Transfected cells were harvested after transfection by trypsinization. After the double staining with fluorescein isothiocyanate-Annexin V and propidium iodide was done by the FITC Annexin V Apoptosis Detection Kit (BD Biosciences, Franklin Lakes, NJ, USA) according to the manufacturer's recommendations. The cells were analyzed with a flow cytometry (FACScan; BD Biosciences) equipped with a Cell Quest software (BD Biosciences). Cells were discriminated into viable cells, dead cells, early apoptotic cells and apoptotic cells, and then the relative ratio of early apoptotic cells were compared with control transfection from each experiment. Cells for cell-cycle analysis were stained with propidium oxide by the CycleTEST PLUS DNA Reagent Kit (BD Biosciences) following the protocol and analyzed by FACScan. The percentage of the cells in G0–G1, S and G2–M phases were counted and compared.

### Xenograft study

Five-week-old female athymic BALB/c mice were maintained under specific pathogen-free conditions and manipulated according to protocols approved by the Shanghai Medical Experimental Animal Care Commission. A549 cells transfected with empty pcDNA3.1 vector or pcDNA3.1-PANDAR were harvested at a concentration of 1 × 10^7^ cells/ml. Of the suspending cells, 0.1 ml was subcutaneously injected into either side of the posterior flank of the nude mouse. Tumor volumes were examined every 2 days when the implantations were starting to grow bigger. Tumor volumes and weights were measured every 2 days in mice from the control vector (seven mice) or pcDNA3.1-PANDAR (seven mice) groups, tumor volumes were measured (length × width^2^ × 0.5). Sixteen days after injection, the mice were killed and tumor weights were measured and used for further analysis. The primary tumors were excised and tumor tissues were used to perform qRT-PCR analysis of PANDAR levels and immunostaining analysis of Ki-67 protein expression.

### ChIP assays

ChIP assays were performed using EZ-CHIP KIT according to the manufacturer's instruction (Millipore). p53 antibody was from Santa Cruz Biotechnology. NF-YA antibody was obtained from Abcam. The ChIP primer sequences were listed in Supplementary Table S1. Quantification of immunoprecipitated DNA was performed using qPCR with SYBR Green Mix (Takara). ChIP data were calculated as a percentage relative to the input DNA by the equation 2^[Input Ct- Target Ct]^ × 0.1 × 100.

### RIP assays

RIP experiments were performed using a Magna RIP™ RNA-Binding Protein Immunoprecipitation Kit (Millipore) according to the manufacturer's instructions. Antibody for RIP assays of NF-YA were from Abcam.

### Subcellular fractionation location

The separation of the nuclear and cytosolic fractions was performed using the PARIS Kit (Life Technologies, Carlsbad, CA, USA) according to the manufacturer's instructions.

### Immunohistochemistry (IHC)

The primary tumors were immunostained for Ki-67 as previously described.^13^

### Statistical analysis

All statistical analyses were performed using SPSS 20.0 software (IBM, SPSS, Armonk, NY, USA). The significance of differences between groups was estimated by Student's *t*-test and χ2 test as appropriate. OS rates were calculated by the Kaplan–Meier method with the log-rank test applied for comparison. Survival data were evaluated using univariate and multivariate Cox proportional hazards model. Variables with a value of *P*<0.05 in univariate analysis were used in subsequent multivariate analysis on the basis of Cox regression analyses. Two-sided *P*-values were calculated, and a probability level of 0.05 was chosen for statistical significance.

## Figures and Tables

**Table 1:** Univariate and multivariate analysis of clinico-pathological factors for overall survival in 140 patients with NSCLC

**Risk factors**	**Univariate analysis**			**Multivariate analysis**		
	HR*	***P*-value**	**95% CI**	**HR**	***P*-value**	**95% CI**
PANDAR expression	0.557	0.001**	0.389∼0.795	0.652	0.015*	0.462∼0.921
Histological grade (low, middle or high)	1.863	0.004**	1.225∼2.833	1.980	0.004**	1.246∼3.144
TNM stage (I/II, III/IV)	3.686	<0.001**	2.380∼5.708	3.448	<0.001**	2.077∼5.723
N (N0, N1 or above)	1.600	0.027*	1.056∼2.424	0.854	0.532	0.520∼1.402
Histological classification (SCC, AD or another)	1.060	0.782	0.702∼1.600			
Age (≤60, >60)	1.075	0.732	0.712∼1.622			
Tumor size (≤3 cm, >3 cm)	1.530	0.071	0.964∼2.428			
History of smoking (ever, never)	1.093	0.673	0.724∼1.650			
Sex (male, female)	1.406	0.116	0.919∼2.151			

Abbreviation: HR, hazard ratio. *P<0.05, **P<0.01

**Table 2:** The clinico-pathological factors of NSCLC patients

**Clinical factors**	**Number of cases**	**(%) of patients**
*Sex*		
male	78	55.7
female	62	44.3
		
*Age*		
≤60	67	47.9
>60	73	52.1
		
*Histological grade*		
high	26	18.6
middle	43	30.7
middle to low	23	16.4
low	45	32.1
other	3	2.1
		
*Histological classification*		
SCC (squamous cell carcinoma)	72	51.4
AD (adenocarcinoma)	64	45.7
other	4	2.9
		
*Tumor stage*		
I	43	30.7
II	53	37.9
III	38	27.1
IV	6	4.3
		
*Tumor (T)*		
T1	37	26.4
T2	58	41.4
T3	36	25.7
T4	9	6.4
		
*Lymph node metastasis (N)*		
N0	69	49.3
N1	54	38.6
N2	15	10.7
N3	2	1.4
		
*History of smoking*		
Ever	74	52.9
never	66	47.1

## Abbreviations

lncRNA: long noncoding rna
PANDAR: promoter of CDKN1A antisense DNA damage activated RNA
NF-YA: nuclear transcription factor Y, alpha
NSCLC: non-small cell lung carcinoma
Bcl-2: B-cell CLL/lymphoma 2
ChIP: Chromatin Immunoprecipitation
RIP: RNA immunoprecipitation
