# Supplementary figures and images for: Low expression of long noncoding RNA PANDAR predicts a poor prognosis of non-small cell lung cancer and affects cell apoptosis by regulating Bcl-2
Source: Cell Death Dis. 2015 Feb 26;6(2):e1665–. doi: 10.1038/cddis.2015.30 (PMC4669812; doi:10.1038/cddis.2015.30)

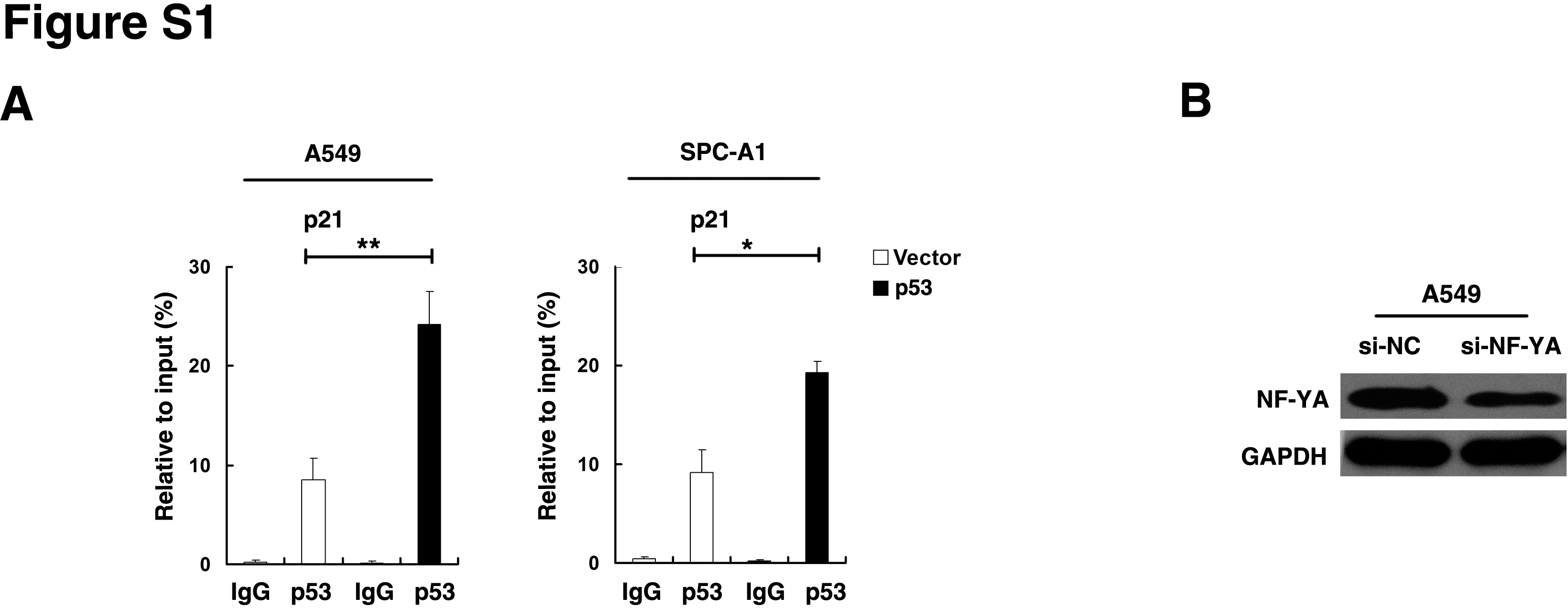

Supplement: Supplementary Figure S1 [file cddis201530x2.tif]
