# Supplementary material for: Low expression of long noncoding RNA PANDAR predicts a poor prognosis of non-small cell lung cancer and affects cell apoptosis by regulating Bcl-2
Source: Cell Death Dis. 2015 Feb 26;6(2):e1665–. doi: 10.1038/cddis.2015.30 (PMC4669812; doi:10.1038/cddis.2015.30)
Supplement: Supplementary Figure Legend [file cddis201530x3.doc]

**Figure S1** (A) The p53 binding at the promoter regions of p21 was assessed by ChIP analysis. (B) Knockdown NF-YA was confirmed by western blotting.
